# A Novel Method for Predicting the Human Inherent Clearance and Its Application in the Study of the Pharmacokinetics and Drug–Drug Interaction between Azidothymidine and Fluconazole Mediated by UGT Enzyme

**DOI:** 10.3390/pharmaceutics13101734

**Published:** 2021-10-19

**Authors:** Yawen Yuan, Jun Zhang, Boyu Fang, Xiaoqiang Xiang, Guo Ma, Shunguo Zhang, Bin Zhu, Weimin Cai

**Affiliations:** 1 Department of Clinical Pharmacy, School of Pharmacy, Fudan University, Shanghai 201203, China; 18111030066@fudan.edu.cn (Y.Y.); 20111030087@fudan.edu.cn (J.Z.); 20211030057@fudan.edu.cn (B.F.); xiangxq@fudan.edu.cn (X.X.); mg0328@fudan.edu.cn (G.M.); 2 Shanghai Children’s Medical Center, Pharmacy Department, Shanghai 200127, China; zhangshunguo@scmc.com.cn; 3 Shanghai BaiO Technology Company, Shanghai 200233, China

**Keywords:** QSAR, metabolic system, clearance, PBPK model, azidothymidine, drug interaction

## Abstract

In order to improve the benefit–risk ratio of pharmacokinetic (PK) research in the early development of new drugs, in silico and in vitro methods were constructed and improved. Models of intrinsic clearance rate (CL_int_) were constructed based on the quantitative structure–activity relationship (QSAR) of 7882 collected compounds. Moreover, a novel in vitro metabolic method, the Bio-PK dynamic metabolic system, was constructed and combined with a physiology-based pharmacokinetic model (PBPK) model to predict the metabolism and the drug–drug interaction (DDI) of azidothymidine (AZT) and fluconazole (FCZ) mediated by the phase II metabolic enzyme UDP-glycosyltransferase (UGT) in humans. Compared with the QSAR models reported previously, the goodness of fit of our CL_int_ model was slightly improved (determination coefficient (R^2^) = 0.58 vs. 0.25–0.45). Meanwhile, compared with the predicted clearance of 61.96 L/h (fold error: 2.95–3.13) using CL_int_ (8 µL/min/mg) from traditional microsomal experiment, the predicted clearance using CL_int_ (25 μL/min/mg) from Bio-PK system was increased to 143.26 L/h (fold error: 1.27–1.36). The predicted C_max_ and AUC (the area under the concentration–time curve) ratio were 1.32 and 1.84 (fold error: 1.36 and 1.05) in a DDI study with an inhibition coefficient (Ki) of 13.97 μM from the Bio-PK system. The results indicate that the Bio-PK system more truly reflects the dynamic metabolism and DDI of AZT and FCZ in the body. In summary, the novel in silico and in vitro method may provide new ideas for the optimization of drug metabolism and DDI research methods in early drug development.

## 1. Introduction

The pharmacokinetic (PK) properties of a therapeutic agent play an important role in drug discovery and development [1]. Good in vitro activity cannot be extrapolated to good in vivo efficacy unless a drug candidate possesses sufficient bioavailability and desirable duration of action. Similarly, the toxicity of the drug in the body is related to its excessive concentration in vivo or specific accumulation in the tissues. As reported, about 40% of candidate compounds were eliminated due to unsatisfactory clinical PK properties in the development of new drugs in the 1980s [2]. In recent years, PK profiling has been routinely implemented in pharmaceutical industries as early as the preclinical optimization process of candidate compounds. More and more attention has been paid to the research and exploration of the PK properties of compounds during the development of new drugs [3,4].

Currently, a number of in vitro methods describing the ADME (absorption, distribution, metabolism and excretion) property of novel drugs have been developed to achieve high-throughput screening in the early drug development process [5]. Traditional in vitro methods for metabolism study include microsomal experiments, liver cell models, recombinase experiments, liver S9 (post-mitochondrial supernatant) models, cytosol experiments and liver slice models [6,7,8,9]. These in vitro metabolism experiments can be used for metabolic stability high-throughput screening and prediction of metabolic clearance (CL) and DDI in the early development of new drugs, thereby facilitating the structural optimization of compounds. However, traditional in vitro metabolic experiments still have some limitations, such as the lack of microenvironment and the inability to truly simulate the 3D environment in the body [10], which may lead to an underestimation of metabolic CL. For example, a lot of attempts were made to improve the traditional in vitro metabolism experiment, but few reflected the true extent of the glucuronidation of the classic UGT2B7 substrate AZT in vivo [11,12,13,14,15,16,17,18,19].

Meanwhile, our previous work was focused on trying to improve the microsomal incubation testing [20,21]. A microsome-hydrogel encapsulation system was developed, and a dynamic perfusion device was introduced to form a Bio-PK dynamic system, simulating the dynamic metabolism and 3D environment in the body [22]. The microsome-hydrogel encapsulation system avoids the direct contact between the microsomes and the drugs in the traditional microsome experiment, thereby prolonging the co-incubation time of the drug and the microsomes [23]. A new research tool of “drug–metabolism–toxicity” system was constructed based on the microsome-hydrogel encapsulation system, and it was successfully applied to the early screening of antitumor candidate compounds and the study of antitumor effect of baicalein after metabolism [20,23,24]. Moreover, the in vivo–in vitro correlation of Bio-PK dynamic system was verified through CYP probe substrates, and it was found that compared with traditional microsome incubation testing, the fitting results of the PBPK model combined with the Bio-PK dynamic metabolism system were closer to the true disposal situation of drugs in vivo [21,25]. However, the applicability of the Bio-PK dynamic system in other metabolic pathways (such as phase II metabolism) and the DDI study still need to be further explored.

In addition to in vitro methods, in silico methods have also been developed in recent years, such as the construction of models based on QSAR to predict the biological activities of candidate compounds, for rapid preliminary screening in the process of drug development [26,27]. Since the 1990s, with the rapid development of bioinformatics and computer technology, more and more in silico approaches have been applied during the various processes of new drug development, including target prediction, ADME screening, structural design and structural optimization [28,29,30,31]. However, due to limited data records and resources, deep learning was difficult to implement and few global QSAR models were built, which impeded the extensive popularization and application of these models [32]. For example, Pirovano et al. built QSAR models using a linear model for metabolic CL prediction based on no more than 250 compounds (hepatocytes: 118 compounds, microsomes: 115 compounds) [33].

In order to improve the efficiency of the early screening of new drugs and to increase the benefit–risk ratio of drug research and development, we first tried to build global QSAR models of CL_int_ using a variety of computer learning methods based on a large number of collected CL_int_ records (8195). At the same time, the Bio-PK equipment was further improved by installing a dialysis syringe pump device to facilitate the real-time quantitative detection of compounds in the hydrogel. In addition, the improved Bio-PK system was first applied to predict the metabolism and DDI of AZT and FCZ mediated by the UGT enzyme in humans.

## 2. Materials and Methods

### 2.1. Construction of CL_int_ QSAR Model

#### 2.1.1. Data Collection and Processing

In all, 8195 CL_int_ records were collected from the literature (2000–2018) and databases such as ChEMBL http://www.ebi.ac.uk/chembl/, accessed on 17 October 2019) and DrugBank (http://redpoll.pharmacy.ualberta.ca/drugbank/, accessed on 17 October 2019) to form the initial data set [27,34,35,36,37]. The unit of CL_int_ was unified as “μL/min/mg”. The data were processed as follows. If the collected CL_int_ was a range value, its average value was calculated as the input value. The repeated records were carefully checked and removed according to the molecular name, structure code and characteristics of the drug, and a more reliable CL_int_ value was retained.

#### 2.1.2. QSAR Model Construction

First, the compound code set was converted into a structure set in sdf format through Open Babel (v.2.4.1) [38]. Second, a descriptor set containing 855 descriptors was constructed based on the compound structure set using the software PaDEL-Descriptor (v.2.21) [39]. Moreover, in order to further simplify the QSAR model, the descriptors were selected according to the relevance and importance of the features to the CL_int_. The 60 best features were selected using mutual information for the following model construction. Moreover, some excessive CL_int_ values were excluded and CL_int_ records in the range of 0.01–1000 μL/min/mg were selected. Finally, a data set including 7882 CL_int_ records was established (see Supplementary Table S1).

The data set was divided into a training set and a test set according to a ratio of 8:2. A novel concept of classification regression model was proposed during the model construction: the training set was further divided into three groups depending on the CL_int_ values and the range of each group was 0–10, 10–100, and 100–1000 μL/min/mg. First, a hierarchical classification model was built to predict which group the compound belonged to using a boosting tree (BT) algorithm [40]. Then, classification regression models were constructed to predict CL_int_ for compounds in each group using four machine learning techniques such as random forest (RF), adaptive boosting (ADA), xgboost (XG) and lightgbm (LGB) [41,42,43,44]. Moreover, average predicted values of the four models were calculated to build an average classification consensus model [45,46].

#### 2.1.3. QSAR Model Evaluation

A fivefold cross-validation was applied to calibrate the training set. Four parameters such as R^2^, correlation coefficient (r), mean absolute error (MAE) and root mean square error (RMSE) were calculated to evaluate the comprehensive performance of the models. R^2^ and r represent the goodness of fit. MAE and RMSE are used to evaluate the prediction errors. The lower the values of MAE and RMSE, and the closer R^2^ and r are to 1, the better the comprehensive performance of the model. The calculation formulae are as follows:

(1) $$R^{2}=1-\frac{\sum_{i}{\left(x_{i}-y_{i}\right)}^{2}}{\sum_{i}{\left(x_{i}-\bar{x}\right)}^{2}}$$

(2) $$r=\frac{\sum_{1}^{N}\left(x_{i}-\bar{x}\right)(y_{i}-\bar{y})}{\sqrt{\sum_{1}^{N}{\left(x_{i}-\bar{x}\right)}^{2}\sum_{1}^{N}{\left(y_{i}-\bar{y}\right)}^{2}}}$$

(3) $$\mathrm{MAE}=\frac{\sum_{1}^{N}\left|x_{i}-y_{i}\right|}{N}$$

(4) $$\mathrm{RMSE}=\sqrt{\frac{\sum_{1}^{N}{\left(x_{i}-y_{i}\right)}^{2}}{N}}$$

where *x_i_* and *y_i_* are the observation concentration and the prediction concentration, respectively; *x* and *y* are the average of the observation and prediction values, respectively; *N* is the number of data points.

### 2.2. Bio-PK Metabolic System

#### 2.2.1. Standards and Reagents

Uridine 5′-diphosphoglucuronic acid trisodium salt (UDPGA Na), Tris buffer (PH = 7.5) and human liver microsomes (HLM) were purchased from Sigma-Aldrich (St. Louis, MO, USA). AZT, FCZ, azidothymidine-glucuronide (AZTG), PBS buffer and potassium hydroxide were purchased from Melone Pharmaceutical Co., Ltd. (Dalian, China). Alamethicin, bovine serum albumin (BSA) and magnesium chloride were purchased from J&K Scientific Ltd. (Beijing, China). Perchloric acid (HCLO_4_) was purchased from the hazardous compound platform of Fudan University. Distilled and deionized water was purified from Milli-Q water system (Millipore, Molsheim, France). Other reagents were of analytical grade.

#### 2.2.2. Preparation of Human Liver Microsome-Hydrogel (HLM-Gel) System

The synthesis of microsomal hydrogel was as previously described [20,21]. Due to the thermosensitive property of the hydrogel, HLM-Gel was prepared by immersing the hydrogel in the HLM solution at 4 °C.

#### 2.2.3. HPLC Analysis Method

The HPLC analysis was performed on an Agilent 1260 infinity (G7117C, Agilent, 5301 Stevens Greek Blvd., Santa Clara, CA, USA) coupled to a UV/Vis detector. The detection wavelength was 267 nm. A ZORBAX Eclipse XDB-C18 column (2.1 × 150 mm; 5 μm) (Agilent, Santa Clara, CA, USA) was used for separation with a flow rate at 1 mL/min, and the injection volume was set to 10 μL. The column temperature was kept at 25 °C. The mobile phases comprised (A) 0.2% acetic acid in 100% water and (B) acetonitrile. Elution was carried out by the gradient method as follows: 5% B kept for 2.0 min, followed by a linear increase to 20% B during 1.5 min and maintained at 20% B for 2.0 min, then linearly decreased to 5% B in 0.5 min and maintained for 1 min. The intra-day precision and accuracy were assessed by testing the QC (quality control) samples (AZT: 15, 400 and 1500 μM; AZTG: 1.5, 25 and 150 μM; FCZ: 75, 1500 and 8000 μM) each day, and the inter-day precision and accuracy were estimated by analyzing the QC samples over three consecutive days. Five samples were determined in each concentration level. The requirements were as follows: the coefficient of variation (CV%) should not exceed 15%, and the relative error (RE%) should be within 85–115%.

#### 2.2.4. Microsomal Incubation Assay

An incubation mixture containing 40 mM MgCl_2_, 250 μg/mL alamethicin and Tris buffer was kept in ice for 30 min with HLM or HLM-Gel to activate the microsomes. The total volume of the incubation system was 300 μL. Then, 20% BSA and AZT were added to the activated microsomes in a 37 °C shaking water bath for 5 min. After pre-incubation, UDPGA (5 mM) was added in the incubation mixture to start the reaction. Next, 75% HCLO_4_ was added to terminate the reaction. After vortexing, the reaction solution was centrifuged (13,300 r/min × 10 min) and the supernatant was transferred to the Eppendorf tube. KOH (4 M) was added to adjust the pH. After centrifugation at the same speed for 5 min, the supernatant was aspirated for HPLC analysis. Moreover, in order to investigate the influence of incubation time, the reaction was terminated by adding 75% HCLO_4_ at 30 min, 1 h, 2 h, 4 h, 8 h, 12 h, 24 h and 48 h, respectively. According the incubation results, the incubation time was set to 1 and 8 h for HLM and HLM-Gel, respectively, and the final concentration of AZT in the microsomal metabolic reaction was set to 50, 100, 250, 400, 600, 800, 1000 and 2000 μM.

#### 2.2.5. Bio-PK Metabolic System Construction

**Figure 1:**
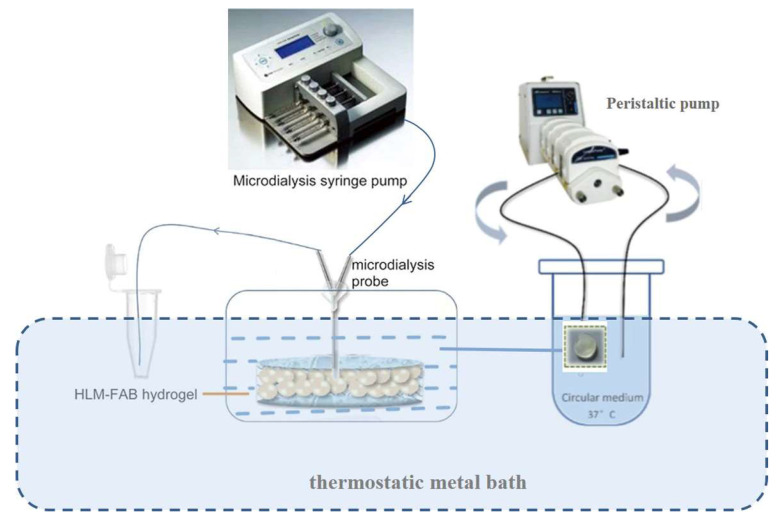
The schematic of the Bio-PK system.

The novel Bio-PK metabolic system was composed of a peristaltic pump, a dialysis pump, an incubation chamber, a thermostatic metal bath and a recirculation pipeline (Figure 1). The incubation system was added in the incubation chamber, in which the metabolic incubation process took place. The peristaltic pump enabled the incubation system to achieve dynamic circulation. Microdialysis technology using the dialysis pump was conducive to the quantitative sampling of drug concentration in the HLM-Gel. A thermostatic metal bath kept the incubation mixture at 37 °C. Recirculation piping connected the various components.

#### 2.2.6. Metabolism of AZT in the Bio-PK Metabolic System

The metabolism of AZT in the Bio-PK system at final concentrations of 500, 1000 and 2500 μM was investigated, respectively. The concentration of the reagents in the incubation system was the same as that in the “Microsomal Incubation Assay”, while the capacity was magnified. The total volume of the Bio-PK incubation system and HLM-Gel was 2000 and 200 μL, respectively. After the Bio-PK metabolic system construction, the perfusion rate was set to 1.6 μL/min, and the peristaltic pump was turned on at speed of 10 mL/min. The circulating reaction solution and dialysate were collected at 0, 0.5, 1, 1.5, 2, 3, 4, 6 and 8 h, respectively. The circulating solution sample and the dialysate were taken for HPLC analysis after pretreatment.

According to the amount change of the substrate AZT and the metabolite AZTG over time in the circulating system and the hydrogel, a mathematical model was established by MATLAB (MathWorks, Inc., Natick, MA, USA) to obtain CL_int_ via fitting the metabolism and cyclic dynamic processes of the compounds in the system. The mathematical formulae are shown below. The determinations of microdialysis recovery, diffusion coefficient and free fraction (*f*_u_) are introduced in Supplementary Table S2, Figure S1 and Table S3, respectively.

$$\frac{dC_{m}\left(t\right)V_{m}}{dt}=D\left(C_{g}\left(t\right)-f_{m}C_{m}\left(t\right)\right)+\left(1-\gamma \right)QC_{m}\left(t-\tau_{1}\right)-\gamma QC_{u}\left(t\right)-QC_{m}\left(t\right)$$

$$\frac{dC_{g}\left(t\right)V_{g}}{dt}=-D\left(C_{g}\left(t\right)-f_{m}C_{m}\left(t\right)\right)+\gamma QC_{m}\left(t-\tau_{1}\right)-CL_{g}C_{g}\left(t\right)-\gamma QC_{u}\left(t-\tau_{2}\right)$$

$$C_{u}\left(t\right)=C_{g}\left(t+\tau_{2}\right)-CL_{g}C_{g}\left(t+\tau_{2}\right)\tau_{2}/V_{g}$$

$$\frac{A_{MTB}\left(t\right)}{dt}=CL_{g}C_{g}\left(t\right)$$

*V_m_* and *V_g_* are the volume of circulating fluid and hydrogel, respectively; *C_m_*, *C_g_* and *C_u_* are the concentration of the substrate in the circulating fluid, the concentration of the substrate in the hydrogel and the concentration of the substrate flowing out of the hydrogel, respectively; *A_MTB_* is the amount of metabolites produced; *Q* is the flow rate of circulating perfusion; *D* is the diffusion coefficient; *f_m_* is the free fraction; *CL_g_* is the clearance rate of the substrate in the hydrogel; *γ* is the part of the substrate passing through the hydrogel from the port of the pipeline; *τ*_1_ and *τ*_2_ are the time taken for the circulation of the substrate and the time the substrate has passed in the hydrogel, respectively.

#### 2.2.7. Interaction of AZT and FCZ in the Bio-PK Metabolic System

Based on the establishment of AZT metabolism in the Bio-PK system, the inhibitor FCZ was added in the Bio-PK system to investigate the interaction of AZT and FCZ. The inhibitory effect of FCZ at three concentration levels of 500, 1000 and 2500 μM on AZT (500 μM) was investigated, respectively, in the Bio-PK system. The incubation method was the same as that in the “Metabolism of AZT in the Bio-PK Metabolic System”.

Similarly, according to the amount change of AZT, AZTG and FCZ over time in the circulating system and the hydrogel, a mathematical model was established by MATLAB to obtain Ki by fitting the metabolism, inhibition and cyclic dynamic processes of the compounds in the system. The mathematical formulae are as shown below:

$$\frac{dC_{m}\left(t\right)V_{m}}{dt}=D\left(C_{g}\left(t\right)-f_{m}C_{m}\left(t\right)\right)+\left(1-\gamma \right)QC_{m}\left(t-\tau_{1}\right)-\gamma QC_{u}\left(t\right)-QC_{m}\left(t\right)$$

$$\frac{dC_{g}\left(t\right)V_{g}}{dt}=-D\left(C_{g}\left(t\right)-f_{m}C_{m}\left(t\right)\right)+\gamma QC_{m}\left(t-\tau_{1}\right)-\frac{CL_{g}}{1+Ci_{g}\left(t\right)/Ki}C_{g}\left(t\right)-\gamma QC_{u}\left(t-\tau_{2}\right)$$

$$C_{u}\left(t\right)=C_{g}\left(t+\tau_{2}\right)-\frac{CL_{g}}{1+Ci_{g}\left(t\right)/Ki}C_{g}\left(t+\tau_{2}\right)\tau_{2}/V_{g}$$

$$\frac{A_{MTB}\left(t\right)}{dt}=\frac{CL_{g}}{1+Ci_{g}\left(t\right)/Ki}C_{g}\left(t\right)$$

$$\frac{dCi_{m}\left(t\right)V_{m}}{dt}=Di\left(Ci_{g}\left(t\right)-f_{m}Ci_{m}\left(t\right)\right)+\left(1-\gamma i\right)QC_{im}\left(t-\tau_{1}\right)-\gamma iQCi_{u}\left(t\right)-QCi_{m}\left(t\right)$$

$$\frac{dCi_{g}\left(t\right)V_{g}}{dt}=-Di\left(Ci_{g}\left(t\right)-f_{m}C_{im}\left(t\right)\right)+\gamma iQCi_{m}\left(t-\tau_{1}\right)-CLi_{g}Ci_{g}\left(t\right)-\gamma QCi_{u}\left(t-\tau_{2}\right)$$

$$Ci_{u}\left(t\right)=C_{ig}\left(t+\tau_{2}\right)-CLi_{g}Ci_{g}\left(t+\tau_{2}\right)\tau_{2}/V_{g}$$

$$\frac{Ai_{m}(0)-Ai_{m}\left(t\right)-Ai_{g}\left(t\right)}{dt}=CLi_{g}C_{ig}\left(t\right).\;$$

*Ci_m_* and *Ci_g_* represent the concentration of the inhibitor in the circulating fluid and hydrogel, respectively; *Ci_u_*, the concentration of the inhibitor flowing out of the hydrogel; *Ai_m_* and *Ai_g_*, the amount of the inhibitor in the circulating fluid and in the hydrogel; *Di*, the diffusion coefficient of the inhibitor; *CLig*, the clearance rate of the inhibitor in the hydrogel; *γi*, the part of the inhibitor that passes through the hydrogel from the port of the pipeline; the others are the same as those in metabolism of AZT in the Bio-PK metabolic system.

#### 2.2.8. PBPK Model Construction

PBPK models were constructed with physical chemistry and ADME parameters, using Simcyp (Simcyp Version 16, Sheffield, UK). Healthy humans were chosen as simulation objects. The physiological conditions and parameters we applied were the default parameters of the healthy humans in the software of Simcyp. The input CL_int_ and Ki were obtained from the experiments described above. The other features parameterized in PBPK models were collected from the literature and databases and tabulated in Supplementary Table S4. First, the AZT metabolic model was constructed, and the simulated PK results of AZT in humans after a single oral administration of 200 mg were evaluated. Secondly, the PBPK model of FCZ was further constructed. And then, the FCZ model was verified and optimized through the PK data of FCZ in human following oral administration at 50, 100, 150 and 200 mg reported from literature. Finally, in order to simulate the DDI process between AZT and FCZ in the human body, the FCZ model was integrated into the AZT metabolic model by introducing Ki.

In order to verify the prediction accuracy of PBPK models, predicted PK parameters (AUC, peak plasma concentration (C_max_) and time to reach C_max_ (T_max_)) were compared with the observed ones, and fold error was calculated to measure the deviation. The fold error is the ratio between the predicted PK parameters and the corresponding observed one. It is currently believed that the simulation is acceptable if the fold error is less than the threshold of 2. The equations for calculating fold error are shown below.

fold error = $\frac{\mathrm{Observed}\;\mathrm{parameter}}{\mathrm{Predicted}\;\mathrm{parameter}}$; if observed > predicted

fold error = $\frac{\mathrm{Predicted}\;\mathrm{parameter}}{\mathrm{Observed}\;\mathrm{parameter}}$; if predicted > observed

## 3. Results

### 3.1. QSAR Model of CL_int_

**Figure 2:**
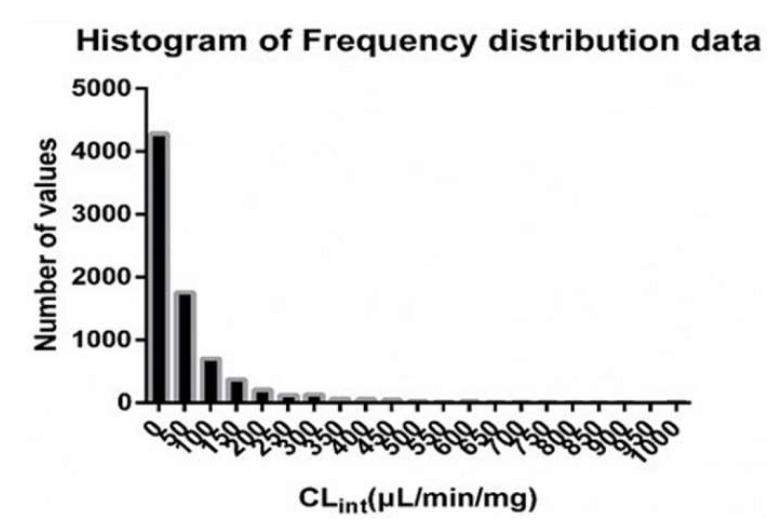
Histogram of frequency distribution of CL_int_ data.

The data distribution of CL_int_ was analyzed and the results are shown in Figure 2. The predicted results of the models are shown in Table 1. The results showed that, compared with other models, the overall performance of the consensus model was better with lower MAE and RMSE (MAE: 33.52 vs. 32.95–37.28 μL/min/mg, RMSE: 83.59 vs. 84.28–88.70 μL/min/mg) and larger R^2^ and r (R^2^: 0.50 vs. 0.44–0.50, r: 0.71 vs. 0.68–0.71). The comparison between the observed CL_int_ and the predicted CL_int_ is shown in Supplementary Figure S2.

### 3.2. HPLC Method Validation

**Figure 3:**
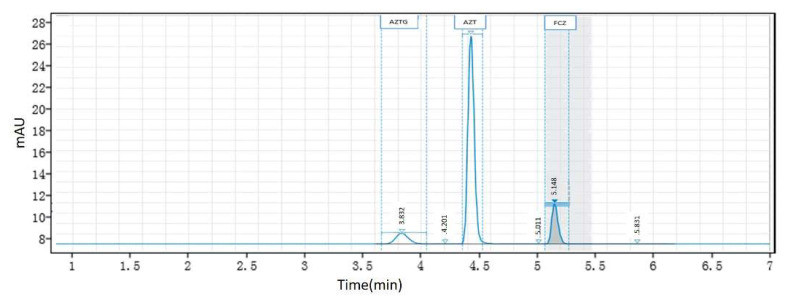
Representative chromatogram of LLOQ sample obtained via spiking AZT, AZTG and FCZ in microsomes.

A representative chromatogram of the LLOQ (the lower limit of quantification) sample, obtained via spiking AZT, AZTG and FCZ in microsomes, is shown in Figure 3. The retention times of AZT, AZTG and FCZ were 4.37, 3.83 and 5.15 min, respectively. The calibration curves were plotted as the response versus the analyte concentration. The calibration curves of eight points were found to be linear over the concentration range of 10–2000 μM of AZT, 1–200 μM of AZTG and 50–10,000 μM of FCZ in Table 2. The correlation coefficients were greater than 0.99 for all curves. For within- and between-day precisions, CV% values calculated for all the tested levels (*n* = 5) did not exceed 15%. For within- and between-day accuracies, the value of RE% ranged from 85% to 115%. The results indicate that this method performs with adequate reliability and reproducibility within the analytical range.

### 3.3. Incubation Time of HLM-Gel System

**Figure 4:**
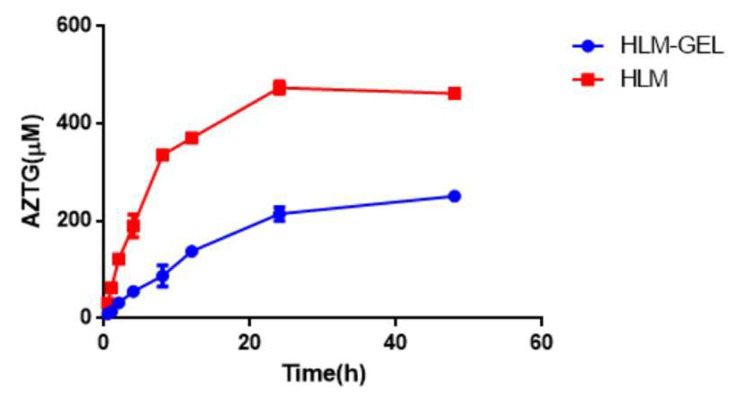
Metabolism of AZT in HLM and HLM-Gel.

The metabolism of AZT within 48 h after the microsomes encapsulated in the hydrogel is shown in Figure 4. The results indicated that there was a delay in metabolism after the microsomes were encapsulated in the hydrogel. The amount of the produced AZTG basically reached equilibrium within 48 h in the traditional HLM experiment, while it still showed a slight upward trend in the HLM-Gel experiment. This indicates that it takes a certain period of time for the drugs to diffuse into the hydrogel, before they contact with the microsomes to undergo the metabolic reaction in the HLM-Gel system. Moreover, the microsomes encapsulated by the hydrogel may retain their activity for a longer time. Therefore, the incubation time of the HLM-Gel experiment should be prolonged compared with that of the traditional HLM experiments. As shown in Figure 4, the formation of AZTG increased linearly within 0.5–8 h in a traditional HLM experiment, while it increased linearly within 24 h in the HLM-Gel experiment. The reaction times of HLM experiments and HLM-Gel incubation experiments were set to 1 and 8 h, respectively.

### 3.4. Metabolism via HLM and HLM-Gel

**Figure 5:**
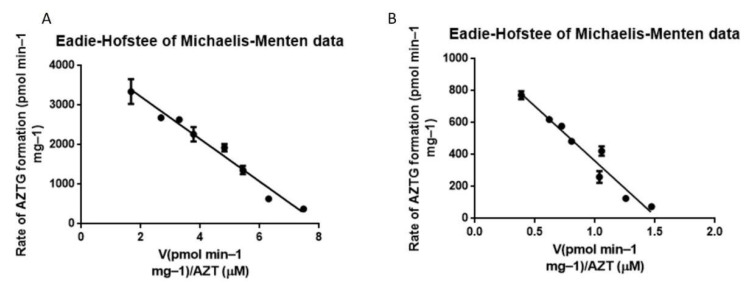
Metabolism kinetics of AZT in HLM (**A**) and HLM-Gel (**B**).

The kinetic profiles of the formation of AZTG via HLM and HLM-Gel followed Michaelis–Menten kinetics as shown in Figure 5. The *V*_max_, *K*_m_ and CL_int_ are listed in Table 3. The results showed that the in vitro metabolic parameters obtained by the traditional HLM experiment were within the range of the parameters reported in the literature (8.1, which is within 0.38–12.81 μL/min/mg protein, see Supplementary Table S5), indicating that the HLM incubation system and the experimental operations were reasonable. Compared with the HLM experiment, the CL_int_ value obtained through the HLM-Gel experiment dropped by nearly four times (8.1 vs. 1.52 μL/min/mg protein). Therefore, it is considered unreasonable to directly fit the kinetic data from the HLM-Gel experiment using the traditional Michaelis–Menten equations to obtain CL_int_, without considering the influence of diffusion. This was also consistent with the results of the previous studies [20,21].

### 3.5. Bio-PK Metabolic System

**Figure 6:**
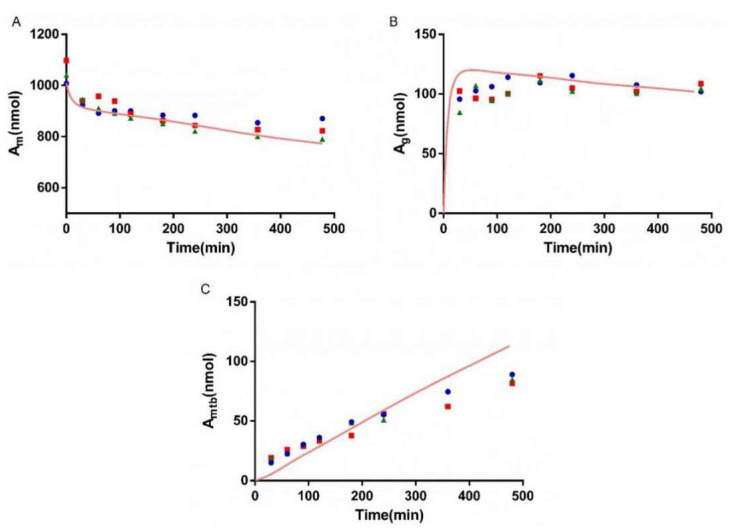
The simulated amount–time curve of AZT in circulating mixture (**A**), hydrogel (**B**) and the simulated amount–time curve of AZTG (**C**) produced in the system at 500 μM. Dots (*n* = 3): experimental data from the Bio-PK system.

**Figure 7:**
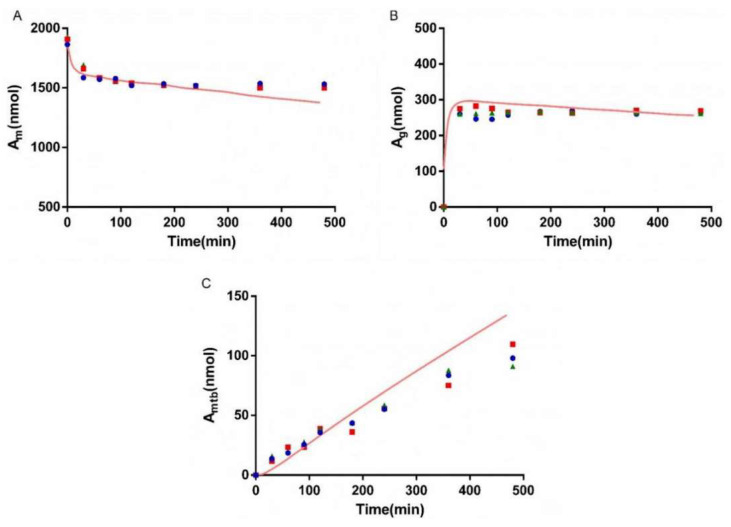
The simulated amount–time curve of AZT in circulating mixture (**A**), hydrogel (**B**) and the simulated amount–time curve of AZTG (**C**) produced in the system at 1 mM. Dots (*n* = 3): experimental data from the Bio-PK system.

**Figure 8:**
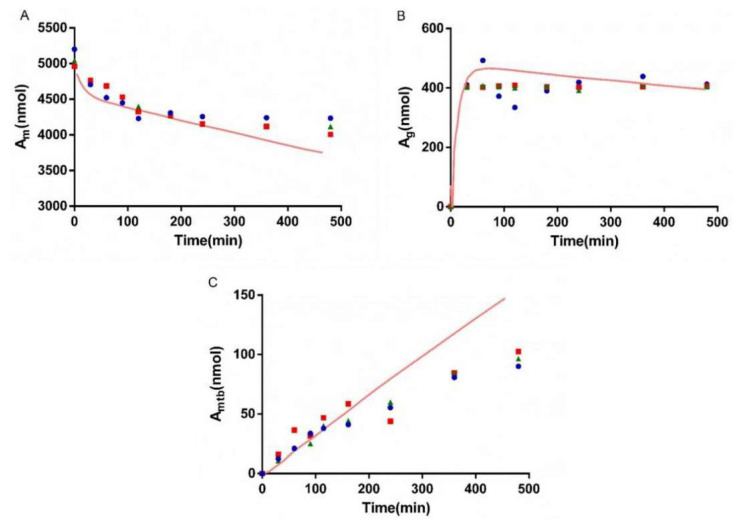
The simulated amount–time curve of AZT in circulating mixture (**A**), hydrogel (**B**) and the simulated amount–time curve of AZTG (**C**) produced in the system at 2.5 mM. Dots (*n* = 3): experimental data from the Bio-PK system.

The metabolism of AZT in the Bio-PK system at three concentration levels (500 μM, 1 mM and 2.5 mM) was investigated, and the CL_g_ was fitted using the MATLAB mathematical model. The fitting curves are shown in Figure 6, Figure 7 and Figure 8. The CL_g_ was divided by the protein concentration of the microsomes to obtain the CL_int_ values, which were 29, 25 and 21 μL/min/mg at 500 μM, 1 mM and 2.5 mM, respectively. The average value was calculated (25 μL/min/mg) and applied as the input value in the PBPK models. In addition, the non-metabolic loss rate of AZT in the system was also estimated based on changes in the amount of AZT and AZTG over time. The results are shown in Table 4. The recovery rates were above 85%, indicating that the non-metabolic loss of AZT in the Bio-PK system was acceptable and reliable.

### 3.6. Interaction Study of AZT and FCZ

**Figure 9:**
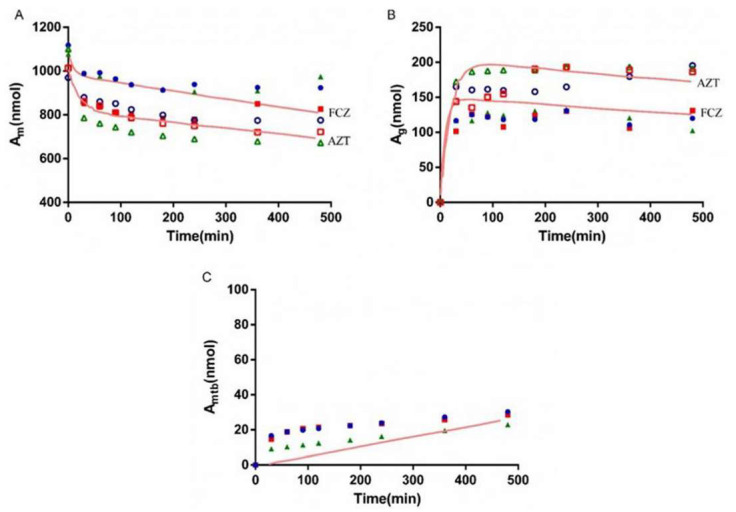
The simulated amount–time curve of AZT and FCZ in circulating mixture (**A**), hydrogel (**B**) and the simulated amount–time curve of AZTG (**C**) produced in the system at 500 μM of AZT and FCZ. Dots (*n* = 3): experimental data from the Bio-PK system.

**Figure 10:**
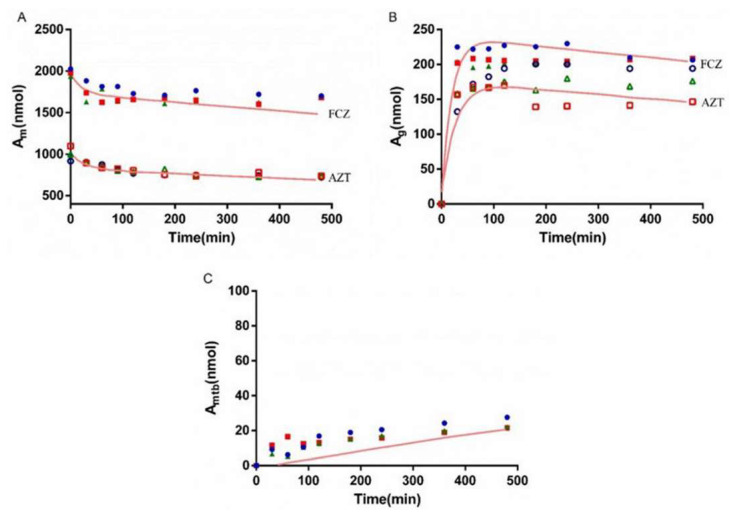
The simulated amount–time curve of AZT and FCZ in circulating mixture (**A**), hydrogel (**B**) and the simulated amount–time curve of AZTG (**C**) produced in the system at 500 μM of AZT and 1 mM of FCZ. Dots (*n* = 3): experimental data from the Bio-PK system.

**Figure 11:**
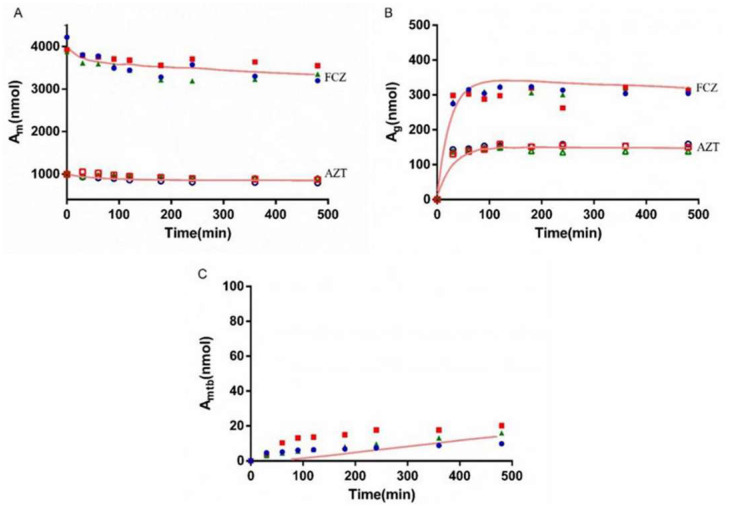
The simulated amount–time curve of AZT and FCZ in circulating mixture (**A**), hydrogel (**B**) and the simulated amount–time curve of AZTG (**C**) produced in the system at 500 μM of AZT and 2 mM of FCZ. Dots (*n* = 3): experimental data from the Bio-PK system.

The inhibitory effects of FCZ at three concentrations (500 μM, 1 mM and 2 mM) on the metabolism of AZT (500 μM) were studied in the Bio-PK system. The inhibition was fitted by MATLAB via mathematical modeling, and the fitting results are shown in Figure 9, Figure 10 and Figure 11. The simulated Ki values were 13.54, 16.9 and 11.46 μM at the FCZ concentration of 500 μM, 1 mM and 2 mM, respectively, and the average value (13.97 μM) was calculated as the input value of the PBPK model. Similarly, the non-metabolic loss rates of AZT and FCZ in the system were estimated based on the changes in the amount of AZT, AZTG and FCZ over time. The results are shown in Table 5. The recovery ranged from 89.99% to 105.58%, indicating that the system was reliable.

### 3.7. Prediction of PK and Interaction Results from PBPK Models

**Figure 12:**
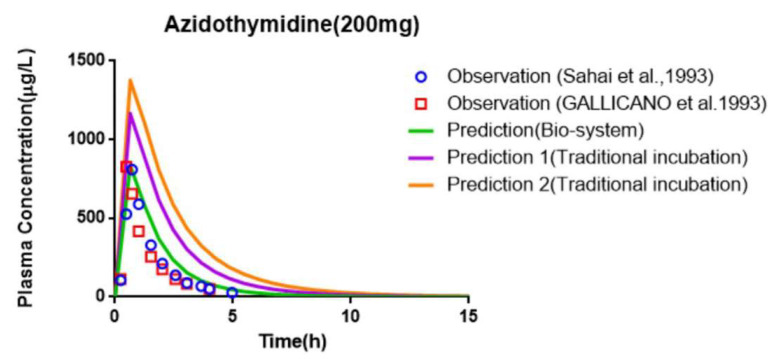
Comparison of the observed plasma concentration–time data with the simulated plasma concentration–time profile of AZT PBPK model in humans at 200 mg.

**Figure 13:**
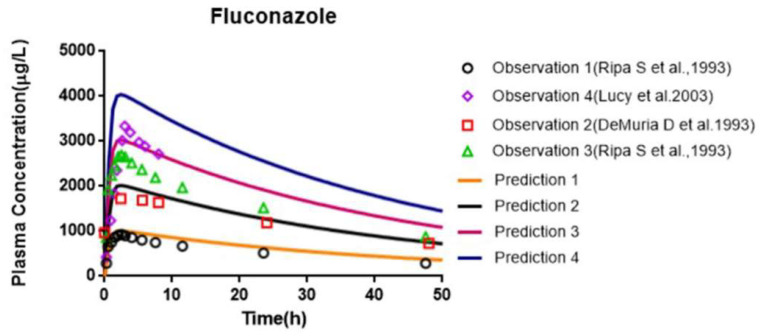
Comparison of the observed plasma concentration–time data with the simulated plasma concentration–time profile of FCZ PBPK model at different doses: prediction 1 at 50 mg, prediction 2 at 100 mg, prediction 3 at 150 mg, prediction 4 at 200 mg.

PBPK models were constructed in healthy humans after a single administration of AZT and FCZ, and the prediction accuracy of the PBPK models is shown in Table 6 and Table 7. The comparison between the predicted plasma concentration–time curves of AZT and FCZ and the corresponding observed plasma concentration points are illustrated in Figure 12 and Figure 13. The PBPK model parameterized with the largest reported CL_int_ value (12.8 µL/min/mg) obtained from the traditional microsomal incubation experiment was constructed, and the predicted PK parameters (AUC, C_max,_ T_max_ and CL) were 2570.08 μg⋅h/L, 1163.65 μg/L, 0.63 h and 84.33 L/h, respectively. The fold errors of AUC and CL were more than 2, indicating the underestimation of the clearance of the traditional microsomal testing. However, for the PBPK model using the average CL_int_ (25 μL/min/mg) obtained from the Bio-system, the predicted CL was increased to 143.26 L/h and the predicted AUC was decreased to 1529.21 μg⋅h/L. Meanwhile, the fold errors of the predicted PK parameters of the PBPK model combined with the Bio-PK system were all within 2.

**Figure 14:**
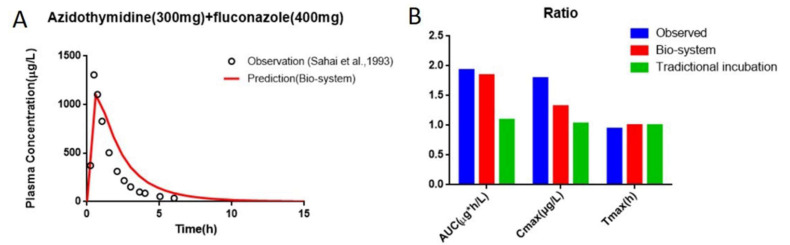
Comparison of the observed plasma concentration–time data with the simulated plasma concentration–time profile of PBPK model of AZT in the presence of FCZ (**A**) and comparison of the observed PK parameter ratios with the predicted PK parameter ratios (**B**).

As shown in Table 7, the fold errors predicted by the FCZ PBPK models at different doses were all less than 2, and the measured FCZ plasma concentration points were close to the predicted PK curve (Figure 13), indicating the reliability of FCZ modeling. The PBPK model of FCZ was further merged into the AZT model by introducing the Ki, obtained from the Bio-PK system, to predict the effect of co-administration of FCZ on the PK of AZT in healthy people. The prediction results are shown in Table 8 and Figure 14. The predicted AUC, C_max_ and T_max_ were 2809.52 μg⋅h/L, 1101.02 μg/L and 0.60 h, and the corresponding predicted PK parameter ratios were 1.84, 1.32 and 1.00, respectively. Meanwhile, the fold errors were all within 2. Moreover, as shown in Figure 14, the predicted PK parameter ratios of the PBPK model parametrized with the Bio-PK system were closer to the observed ratios than those of the PBPK model constructed using Ki (145 μM) [12] from traditional microsomal testing, especially AUC ratio and C_max_ ratio (fold error: 1.05 vs. 1.75, 1.36 vs. 1.73), indicating better simulation accuracy of AZT and FCZ interaction using Bio-PK system.

## 4. Discussion

As shown in Figure 2, the distribution of CL_int_ was extremely uneven. Most compounds were distributed in the lower CL_int_ range (<50 μL/min/mg). The higher the CL_int_ value, the lower the number of compounds was. Meanwhile, the extreme bias of the data distribution would lead to deviations in the model prediction results. We proposed the concept of a classification regression model, trying to balance the distribution in each group and hoping to reduce the prediction error caused by this data distribution bias. In addition, since the standard error (RMSE) or MAE was not as sensitive to large variations of data as r or R^2^, attaching importance to the standard error (RMSE) or MAE was more suitable when evaluating the models [52]. The results showed (Table 1) that, due to the wide range of the CL_int_ values (0.01–1000 μL/min/mg), both MAE and RMSE were relatively large (MAE: 32.95–37.28 μL/min/mg, RMSE: 83.59–88.7 μL/min/mg). Interestingly, ranking QSAR models according to RMSE and MAE gave the same result as ranking them over r or R^2^, i.e., the overall performance of the consensus model was better, which may be caused by the more even distribution of data from the classification regression models. Moreover, compared with previous models reported in the literature, for example, the CL_int_ QSAR model constructed by Ekins [53] with an R^2^ of 0.34, and the model constructed by Aliagas et al. [34] with an R^2^ of 0.25–0.45, the goodness of fit of our CL_int_ model was slightly improved (R^2^ = 0.58). While as shown in Figure S2, there were significantly more prediction points in the area below the correlation line than in the area above it, especially in the larger CL_int_ range (>400 μL/min/mg), indicating that the impact of the uneven distribution of CL_int_ on the prediction accuracy still existed. Therefore, it is believed that the extreme lower value skewed distribution and wide range of the CL_int_ are the main reasons for the prediction error of the models. Moreover, although we had collected as much data as possible, deep learning was still difficult to implement. This limitation has plagued the construction of models for almost all biological activity features. These all pose challenges for future model optimization. In the follow-up research, it may be possible to further refine the data processing including data noise reduction and the group dividing.

Therefore, in vitro metabolism experiments are still indispensable in the preclinical PK research of new drugs. Microsomes are subcellular components prepared by differential centrifugation in organs such as the liver, intestines and kidneys. They are a part of the endoplasmic reticulum of organelles, which express a variety of proteins. Moreover, liver microsomes include complete phase I metabolic enzymes. It can reflect drug metabolism in humans. Currently, many microsomal metabolism models have been widely used in drug in vitro PK studies [11,12,13,14,15,16,17,18,19]. However, although the microsome experiment greatly simplifies the metabolism experiment, microsomes still possess some limitations and deficiencies, such as the lack of microenvironment, the inability to truly simulate the 3D environment in the body, the non-continuous incubation time and the static incubation method. Due to some drawbacks of the in vitro experiments, the CL_int_ of many compounds, such as AZT, was underestimated [10]. It was reported that many adjustments and improvements had been made to the traditional in vitro metabolism experiments of AZT, such as adjusting the composition of the buffer solution, changing pH, adding BSA and so on, to increase the predicted CL_int_ value (Table S5). As shown in Table 6, the maximum value of the CL_int_ value from the traditional microsomal incubation metabolism experiments reported in the literature was parametrized for PBPK modeling, while the prediction results still underestimated the metabolism of AZT in vivo with fold errors of CL and AUC both greater than 2.

In order to more truly reflect the dynamic metabolic process of compounds in the body, a circulation device, the Bio-PK metabolic system, simulating the microfluidic environment in the body was proposed in our lab [20,21,23]. The Bio-PK system improved the limitations of HLM to a certain extent. The microsomes were encapsulated by hydrogel, the incubation took place in a 3D-EP tube, the co-incubation time was extended and a circulation device was introduced to simulate the dynamic circulation in the body. In this study, the Bio-PK equipment was further improved based on previous research results and a dialysis syringe pump device was added to the original equipment to facilitate real-time drug detection. The feasibility of the improved Bio-PK system for use in analyzing the UGT metabolic pathway of AZT and the interaction of AZT and FCZ was explored. However, the simulated quantity–time curves in Figure 6, Figure 7 and Figure 8C did not completely match the experimental values. The simulated metabolism results of AZT in the Bio-PK system within 200 min were consistent with the experimental values, while the predicted production of AZTG within 200–500 min was higher than the experimental data. Similarly, it was found that at a concentration of 2.5 mM, the final amount of metabolites produced in the “Bio-PK” system did not increase significantly compared to the 1 mM concentration of AZT. It is believed that this may be because AZT has completed diffusion and entered the HLM-Gel to reach a stable concentration at 200 min, the microsomal metabolism of AZT in the Bio-PK system may be saturated at 200–500 min, leading to the smaller experimental data. At the same time, the hydrogel may affect the penetration and activation of alamethicin in the microsomes, which can cause a low number of activated microsomes and metabolic saturation. Similarly, the experimental amount of AZTG produced within 200 min was larger than the predicted value (Figure 9, Figure 10 and Figure 11C), this may also be because the inhibitor FCZ needed to diffuse into the hydrogel to play an inhibitory role. After the diffusion of FCZ into the hydrogel to reach a stable concentration, the simulated values were in good agreement with the experimental data within 200–500 min.

As shown in Figure 6, Figure 7 and Figure 8, AZT in the circulation fluid first diffused into the hydrogel and then was metabolized by the microsomes coated in the hydrogel, that is, the amount of AZT in the circulation fluid rapidly dropped to reach equilibrium and then reduced through metabolism. After metabolism, the remaining AZT and the produced AZTG diffused from the hydrogel into the circulating fluid and circulated in the system through the peristaltic pump. Compared with traditional microsome testing, AZT circulated to reach a stable concentration during the incubation process in the Bio-PK system, so as to realize the continuous supply of AZT to the microsomes. Moreover, in this study, CL_g_ was defined as how much volume of hydrogel (μL) containing AZT was completely metabolically eliminated within a unit time (per minute). Since the concentration of AZT in the hydrogel were stable and constant after diffusion, the change in the amount of AZT in the Bio-PK system per minute was constant; that is, AZT was metabolized linearly in the hydrogel (Figure 6, Figure 7 and Figure 8C). Meanwhile, the results indicated that the CL value predicted by the PBPK model, parameterized with the CL_int_ from the Bio-PK system, was close to the actual CL value in vivo with a fold error less than 2 (Table 6). Moreover, the fold errors of other PK parameters were all less than 2, and the observed concentration points were close to the predicted plasma concentration–time curve (Figure 12), indicating the rationality of the construction of the PBPK model.

Since FCZ is rarely metabolized by microsomes, the amount change of FCZ over time in the hydrogel is mainly affected by diffusion [49]. Similarly, AZT and FCZ circulated between the HLM-Gel and circulation fluid through diffusion and peristaltic pumps, thus, increasing the co-incubation time of AZT, FCZ with microsomes (Figure 9, Figure 10 and Figure 11). Therefore, compared with the traditional microsome testing, the Bio-PK system is more suitable for the exploration of slow metabolism and time-dependent DDI in theory. Meanwhile, the results showed that compared with the traditional microsomal incubation testing, the predicted AUC ratio and C_max_ ratio of the PBPK model, parametrized with Ki from the Bio-PK system were increased and closer to the in vivo level (Table 8). The fold errors of the PK parameters were less than 2, indicating the rationality of the PBPK modeling. These results all indicate that the Bio-PK system solves the limitations of traditional microsomal incubation metabolism to a certain extent and may provide a new method and idea for the optimization of in vitro metabolism and DDI methods.

However, the application of the Bio-PK metabolic system is still currently limited to drugs that are mainly metabolized in the liver. For drugs that are mainly eliminated via the metabolism in the gastrointestinal tract and other tissues, the system needs to be further optimized and verified. Similarly, for compounds with complex drug elimination characteristics, such as transporter-mediated excretion, the application of Bio-PK metabolic system and PBPK model needs to be further studied.

## 5. Conclusions

In summary, we established an HPLC method for the simultaneous determination of AZT, AZTG and FCZ, and the HPLC method was successfully applied to the DDI study between AZT and FCZ. It was validated that the HPLC method met the requirements of biological sample analysis and the analytical range of the compounds in DDI study were all within the linear range of the method. Moreover, QSAR models of CL_int_ were successfully constructed based on 7882 records collected from different literature and databases. The fitting correlation was slightly improved compared with that of previous models. However, due to the complexity of the metabolic pathways in the body, the extreme imbalance and extensiveness of the CL_int_ distribution, the prediction accuracy of the CL_int_ model still needs to be further improved. In addition, we further tried to optimize the in vitro metabolism testing and constructed the Bio-PK dynamic metabolism system. The Bio-PK system was combined with the PBPK models and successfully applied to the metabolism and DDI studies of AZT, a substrate of the UGT enzyme, in an attempt to solve the issue of the underestimation of metabolism that would occur in traditional microsomal incubation experiments. Compared with traditional HLM experiments, the fold error of PK parameters predicted by the Bio-PK system combined with the PBPK model was significantly reduced to less than 2, which was closer to the real situation in vivo. In the DDI study, the AUC ratio and C_max_ ratio predicted by the Bio-PK system combined with the PBPK model increased, and the fold error was less than 2, which was closer to the ratio in vivo. These results all show that the Bio-PK system solves the limitations of traditional HLM experiment to a certain extent and may provide new methods and ideas for the optimization of drug metabolism and DDI study in vitro.

## Figures and Tables

**Table 1:** Performance of classification regression models of CL_int_.

Data	Metric	RF	ADA	XG	LGB	Consensus (Average)
5-CV	MAE (μL/min/mg)	35.48	32.87	35.31	36.27	33.32
	RMSE (μL/min/mg)	77.41	83.92	77.08	76.58	75.88
	R^2^	0.56	0.48	0.56	0.57	0.58
	r	0.75	0.71	0.75	0.76	0.76
Test	MAE (μL/min/mg)	35.16	32.95	35.84	37.28	33.52
	RMSE (μL/min/mg)	84.28	88.70	84.66	86.72	83.59
	R^2^	0.50	0.44	0.49	0.47	0.50
	r	0.71	0.68	0.70	0.69	0.71

**Table 2:** Method validation parameters of AZT, AZTG and FCZ.

Parameters	AZT	AZTG	FCZ
Linearity equation (*n* = 8 points)	y = 5.1548x + 21.2189	y = 5.2534x + 3.3663	y = 0.1899x + 4.5826
Correlation coefficient	0.999	1	1
LLOQ (μM)	10	1	50
Intra-day precision (%) * (*n* = 5)	5 ± 2	7.3 ± 1.5	5.6 ± 1.3
Inter-day precision (%) * (*n* = 5)	9.5 ± 3.6	6.8 ± 2.6	3.9 ± 1.9
Intra-day accuracy (%) * (*n* = 5)	104 ± 2	98 ± 1	100.8 ± 2.5
Inter-day accuracy (%) * (*n* = 5)	104 ± 1	99.5 ± 2.2	100.6 ± 0.1

* Mean ± SD.

**Table 3:** In vitro metabolism parameters from HLM and HLM-Gel.

Experimentation	*K*_m_ (μM)	*V*_max_ (pmol/min/mg Microsomal Protein)	CL_int_ (μL/min/mg Protein)
HLM	536	4287	8.1
HLM-Gel	686	1045	1.52

**Table 4:** Recovery rates of AZT in Bio-PK system.

Time (min)	500 μM	1000 μM	2500 μM
30	100.07%	101.32%	103.26%
60	99.78%	98.09%	100.84%
90	99.14%	97.39%	98.16%
120	98.02%	96.38%	94.81%
180	97.64%	96.52%	94.66%
240	96.49%	96.85%	92.90%
360	95.57%	97.98%	93.24%
480	97.15%	98.73%	92.48%

**Table 5:** Recovery rates of AZT and FCZ in Bio-PK system.

Time (min)	AZT			FCZ		
	500-1	500-2	500-3	500	1000	2000
30	101.61%	105.58%	101.46%	96.37%	98.05%	100.35%
60	100.8%	103.56%	101.36%	96.38%	97.5%	100.17%
90	99.54%	99.63%	99.97%	94.02%	95.38%	97%
120	97.32%	98.21%	98%	91.84%	94.69%	95.74%
180	96.35%	95.80%	95.26%	89.99%	93.82%	91.51%
240	95.01%	93.92%	93.51%	91.42%	94.87%	94.38%
360	94.28%	93.92%	93.1%	91.43%	92.85%	92.37%
480	94.82%	92.74%	92.86%	93.48%	94.96%	91.79%

**Table 6:** Prediction results of AZT PBPK model.

AZT (200 mg)		AUC (μg⋅h/L)	C_max_ (μg/L)	T_max_ (h)	CL (L/h)
Observed [47,48]		1020 ± 390	1042 ± 632	0.8 ± 0.3	194 ± 53
		1078 ± 257	1093 ± 679	0.6 ± 0.2	183 ± 53
Traditional incubation	Predicted 1 [12]	2570.08	1163.65	0.63	84.33 ± 36.88
	Fold error	2.52	1.12	1.26	2.30
		2.38	1.06	1.10	2.17
	Predicted 2	3501	1374.73	0.65	61.96 ± 25.23
	Fold error	3.43	1.32	1.24	3.13
		3.25	1.26	1.13	2.95
Bio-system	Predicted	1529.21	833.94	0.6	143.26 ± 67.98
	Fold error	1.5	1.25	1.33	1.36
		1.42	1.31	1.04	1.27

Predicted 1 using the max reported CL_int_ of 12.8 µL/min/mg, predicted 2 using our experimental CL_int_ of 8.0 µL/min/mg.

**Table 7:** Prediction results of FCZ PBPK model.

FCZ	AUC (μg⋅h/L)	C_max_ (μg/L)	T_max_ (h)
Observation 1 [49] (50 mg)	39,810 ± 6460	930 ± 130	2.5
Prediction 1 (50 mg)	45,424.77	1010.38	2.41
Fold error	1.14	1.09	1.04
Observation 2 [50] (100 mg)	99,300	2080	2
Prediction 2 (100 mg)	90,849.33	2020.77	2.41
Fold error	1.09	1.03	1.21
Observation 3 [49] (150 mg)	114,150 ± 19,730	2690 ± 430	2.5
Prediction 3 (150 mg)	136,273.8	3031.14	2.41
Fold error	1.19	1.13	1.04
Observation 4 [51] (200 mg)	207,000 ± 55,700	3580 ± 140	3.2 ± 0.8
Prediction 4 (200 mg)	181,698.11	4041.49	2.41
Fold error	1.14	1.13	1.32

**Table 8:** Prediction results of PBPK model of AZT in the presence of FCZ.

Parameters	AUC (μg⋅h/L)	C_max_ (μg/L)	T_max_ (h)
AZT alone	1020 ± 390	1042 ± 632	0.8 ± 0.3
AZT + FCZ	1965 ± 838	1864 ± 958	0.8 ± 0.3
Ratio	1.93	1.79	0.94
Predicted (AZT alone)	2570.08	1163.65	0.63
Fold error	2.52	1.12	1.26
Predicted(AZT + FCZ)	2812.57	1203.61	0.63
Fold error	1.43	1.55	1.19
Ratio	1.09	1.03	1
Fold error	1.76	1.73	1.06
Predicted(AZT alone)	1529.21	833.94	0.6
Fold error	1.5	1.25	1.33
Predicted (AZT + FCZ)	2809.52	1101.02	0.6
Fold error	1.43	1.69	1.25
Ratio	1.84	1.32	1
Fold error	1.05	1.36	1.06

## Supplementary Materials

The following are available online at https://www.mdpi.com/article/10.3390/pharmaceutics13101734/s1, Figure S1: Diffusion of AZT (A) and FCZ (B) in Bio-PK metabolic system, Figure S2: Comparison of the observed CL_int_ with predicted CL_int_ of the classification regression models, blue line: correlation line, Table S1: The experimental and prediction data of 7882 CL_int_ records collected from the literature (2000–2018) and databases. Table S2: Microdialysis recovery of AZT, AZTG and FCZ, Table S3: The *f*_u_ of AZT and FCZ in the HLM-Gel system, Table S4: Parameters for AZT and FCZ PBPK modeling, Table S5: In vitro metabolism parameters of AZT reported from literature.
