# Supplementary material for: A Novel Method for Predicting the Human Inherent Clearance and Its Application in the Study of the Pharmacokinetics and Drug–Drug Interaction between Azidothymidine and Fluconazole Mediated by UGT Enzyme
Source: Pharmaceutics. 2021 Oct 19;13(10):1734. doi: 10.3390/pharmaceutics13101734 (PMC8538957; doi:10.3390/pharmaceutics13101734)
Supplement: Supplementary file 1 [file pharmaceutics-13-01734-s001.zip › Supplementary Materials/pharmaceutics-1366759-supplementary-done.pdf]

pharmaceutics

Supplementary Materials: A Novel Method for Predicting the Human Inherent Clearance and Its Application in the Study of the Pharmacokinetics and Drug–Drug Interaction between Az-idothymidine and Fluconazole Mediated by UGT Enzyme

Yawen Yuan, Jun Zhang, Boyu Fang, Xiaoqiang Xiang, Guo Ma, Shunguo Zhang, Bin Zhu and Weimin Cai

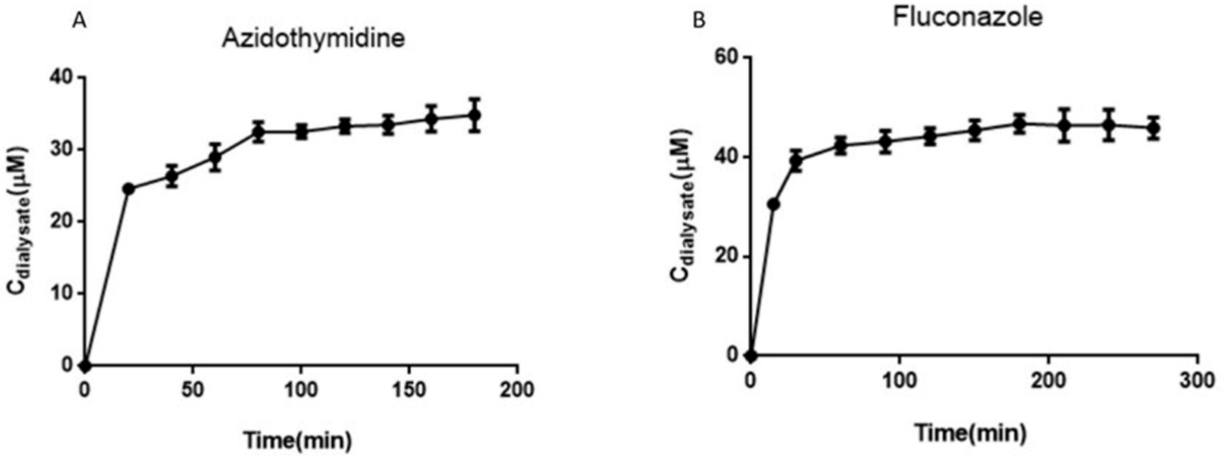

Figure S1. Diffusion of AZT (**A**) and FCZ (**B**) in Bio-PK metabolic system. .

The diffusion coefficients of AZT and FCZ in Bio-PK metabolic system were investigated. The probe was embedded in the HLM-Gel and then perfused with tris buffer at a rate of 1.6 μL/min. After 40 minutes of equilibration, 500 μM AZT and FCZ were added respectively, and the dialysate was collected. The AZT dialysate collection time was set to 20, 40, 60, 80, 100, 120, 140, 160 and 180min respectively, and the FCZ dialysate collection time was set to 15, 30, 60, 90, 120, At 150, 180, 210, 240 and 270 minutes respectively. 75% HCLO4 and 4M KOH were added to adjust the pH and the supernatant was aspirated for HPLC quantitative analysis after centrifugation. The result shows that the concentration of AZT and FCZ in the hydrogel increased rapidly at first, and then reached stability at 80 min and 60 min, respectively. The hydrogel volume is divided by the equilibrium time to obtain the diffusion coefficient of AZT and FCZ, which are 0.0025 mL/min and 0.0033 mL/min, respectively.

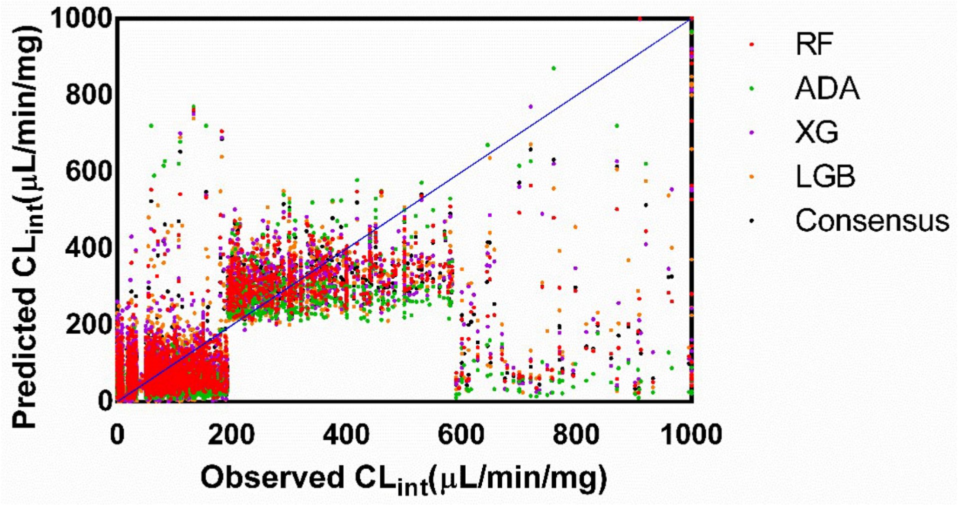

Figure S2. Comparing of the observed CL_{int} with predicted CL_{int} of the classification regression models, blue line: correlation line.
